# Factors associated with anemia and vitamin A deficiency in Brazilian children under 5 years old: *Brazilian National Survey on Child Nutrition* (ENANI-2019)

**DOI:** 10.1590/0102-311XEN194922

**Published:** 2023-09-25

**Authors:** Inês Rugani Ribeiro de Castro, Paula Normando, Dayana Rodrigues Farias, Talita Lelis Berti, Raquel Machado Schincaglia, Pedro Gomes Andrade, Neilane Bertoni, Elisa Maria de Aquino Lacerda, Luiz Antonio dos Anjos, Cristiano Siqueira Boccolini, Marta Citelli dos Reis, Flávia Fioruci Bezerra, Lucia Fatima Campos Pedrosa, Alceu Afonso Jordão, Pedro Israel Cabral de Lira, Gilberto Kac, Brazilian Consortium on Child Nutrition, Letícia B. Vertulli Carneiro

**Affiliations:** 1 Instituto de Nutrição, Universidade do Estado do Rio de Janeiro, Rio de Janeiro, Brasil.; 2 Instituto de Nutrição Josué de Castro, Universidade Federal do Rio de Janeiro, Rio de Janeiro, Brasil.; 3 Divisão de Pesquisa Populacional, Instituto Nacional de Câncer José Alencar Gomes da Silva, Rio de Janeiro, Brasil.; 4 Departamento de Nutrição Social, Universidade Federal Fluminense, Niterói, Brasil.; 5 Instituto de Comunicação e Informação Científica e Tecnológica em Saúde, Fundação Oswaldo Cruz, Rio de Janeiro, Brasil.; 6 Universidade Federal do Rio Grande do Norte, Natal, Brasil.; 7 Faculdade de Medicina de Ribeirão Preto, Universidade de São Paulo, Ribeirão Preto, Brasil.; 8 Centro de Ciências da Saúde, Universidade Federal de Pernambuco, Recife, Brasil.

**Keywords:** Preeschool Child, Micronutrients, Social Determinants of Health, Surveys and Questionnaires, Pré-escolar, Micronutrientes, Determinantes Sociais da Saúde, Inquéritos e Questionários, Preescolar, Micronutrientes, Determinantes Sociales de la Salud, Encuestas y Cuestionarios

## Abstract

Factors associated with anemia and vitamin A deficiency were investigated in 7,716 children 6-59 months of age studied in the *Brazilian National Survey on Child Nutrition* (ENANI-2019). We adopted a hierarchical approach based on a United Nations Children’s Fund (UNICEF) theoretical model with three levels, stratifying by age (6-23; 24-59 months). Prevalence ratio (PR) and 95% confidence interval (95%CI) were estimated. Enabling determinants: a higher prevalence of anemia was observed in children 6-23 months whose mothers had ≤ 7 years of schooling (PR = 1.92; 95%CI: 1.10; 3.34), < 20 years old (PR = 2.47; 95%CI: 1.34; 4.56) or 20-30 years old (PR = 1.95; 95%CI: 1.11; 3.44), mixed-race (PR = 1.57; 95%CI: 1.06; 2.23); and in children 24-59 months in the North Region (PR = 3.11; 95%CI: 1.58; 6.13). A higher prevalence for vitamin A deficiency was observed in children 6-23 months from Central-West (PR = 2.32; 95%CI: 1.33; 4.05), and in children 24-59 months living in the North (PR = 1.96; 95%CI: 1.16; 3.30), South (PR = 3.07; 95%CI: 1.89; 5.01), and Central-West (PR = 1.91; 95%CI: 1.12; 3.25) and whose mothers were 20-34 years (PR = 1.62; 95%CI: 1.11; 2.35). Underlying determinants: the presence of more than one child < 5 years old in the household was associated with a higher prevalence of anemia (PR = 1.61; 95%CI: 1.15; 2.25) and vitamin A deficiency (PR = 1.82; 95%CI: 1.09; 3.05) in children 6-23 months. Immediate determinants: consumption of 1-2 groups of ultra-processed foods in children 24-59 months (PR = 0.44; 95%CI: 0.25; 0.81) and lack of breastfeeding in the day before in children 6-23 months (PR = 0.56; 95%CI: 0.36; 0.95) were associated with lower prevalence of anemia and vitamin A deficiency. Public policies focused on geographically and socially vulnerable groups are needed to promote equity.

## Resumo

Fatores associados a anemia e deficiência de vitamina A foram investigados em 7.716 crianças de 6-59 meses de idade parte da *Estudo Nacional de Alimentação e Nutrição Infantil* (ENANI-2019). Adotamos uma abordagem hierárquica baseada em um modelo teórico do Fundo das Nações Unidas para a Infância (UNICEF) com três níveis estratificados por idade (6-23; 24-59 meses). Foram estimadas razões de prevalência (RP) e intervalos de 95% de confiança (IC95%). Determinantes habilitadores: observamos maior prevalência de anemia em crianças de 6-23 meses de idade cujas mães tinham ≤ 7 anos de escolaridade (RP = 1,92; IC95%: 1,10; 3,34), < 20 anos de idade (RP = 2,47; IC95%: 1,34; 4,56) ou 20-30 anos de idade (RP = 1,95; IC95%: 1,11; 3,44), cor parda (RP = 1,57; IC95%: 1,06; 2,23); e em crianças de 24-59 meses de idade na Região Norte (RP = 3,11; IC95%: 1,58; 6,13). Encontramos maior prevalência de deficiência de vitamina A em crianças de 6-23 meses de idade no Centro-oeste (RP = 2,32; IC95%: 1,33; 4,05) e em crianças de 24-59 meses de idade residentes nas regiões Norte (RP = 1,96; IC95%: 1,16; 3,30), Sul (RP = 3,07; IC95%: 1,89; 5,01) e Centro-oeste (RP = 1,91; IC95%: 1,12; 3,25) cujas mães tinham entre 20-34 anos de idade (RP = 1,62; IC95%: 1,11; 2,35). Determinantes subjacentes: a presença de mais de uma criança < 5 anos de idade no domicílio se associou a maior prevalência de anemia (RP = 1,61; IC95%: 1,15; 2,25) e deficiência de vitamina A (RP = 1,82; IC95%: 1,09; 3,05) em crianças de 6-23 meses de idade. Determinantes imediatos: o consumo de 1-2 grupos de alimentos ultraprocessados em crianças de 24-59 meses de idade (RP = 0,44; IC95%: 0,25; 0,81) e o não aleitamento materno no dia anterior em crianças de 6-23 meses de idade (RP = 0,56; IC95%: 0,36; 0,95) foram associados com a menor prevalência de anemia e deficiência de vitamina A. Políticas públicas focadas em grupos geográfica e socialmente vulneráveis são necessárias para promover equidade.

## Resumen

Los factores asociados con la anemia y la deficiencia de vitamina A se investigaron en 7.716 niños de 6-59 meses de edad que formaban parte del *Estudio Nacional de Alimentación y Nutrición Infantil* (ENANI-2019). Adoptamos un enfoque jerárquico basado en un modelo teórico del Fondo de las Naciones Unidas para la Infancia (UNICEF) con tres niveles estratificados por edad (6-23; 24-59 meses). Se estimaron las razones de prevalencia (RP) y el intervalo de 95% de confianza (IC95%). Factores determinantes: observamos una mayor prevalencia de anemia en los niños de 6-23 meses de edad cuyas madres tenían ≤ 7 años de escolaridad (RP = 1,92; IC95%: 1,10; 3,34), < 20 años de edad (RP = 2,47; IC95%: 1,34; 4,56) o de 20-30 años de edad (RP = 1,95; IC95%: 1,11; 3,44), color pardo (RP = 1,57; IC95%: 1,06; 2,23); y en niños de 24-59 meses edad en la Región Norte (RP = 3,11; IC95%: 1,58; 6,13). Encontramos una mayor prevalencia de deficiencia de vitamina A en los niños de 6-23 meses de edad en el Centro-oeste (RP = 2,32; IC95%: 1,33; 4,05) y en los niños de 24-59 meses de edad que residen en las regiones Norte (RP = 1,96; IC95%: 1,16; 3,30), Sur (RP = 3,07; IC95%: 1,89; 5,01) y Centro-oeste (RP = 1,91; IC95%: 1,12; 3,25) cuyas madres tenían entre 20-34 años de edad (RP = 1,62; IC95%: 1,11; 2,35). Determinantes subyacentes: la presencia de más de un niño < 5 años de edad en el hogar se asoció a la mayor prevalencia de anemia (RP = 1,61; IC95%: 1,15; 2,25) y deficiencia de vitamina A (RP = 1,82; IC95%: 1,09; 3,05) en niños de 6-23 meses de edad. Determinantes inmediatos: el consumo de 1-2 grupos de alimentos ultraprocesados en niños de 24-59 meses de edad (RP = 0,44; IC95%: 0,25; 0,81) y no consumo de leche materno el día anterior en niños de 6-23 meses de edad (RP = 0,56; IC95%: 0,36; 0,95) se asociaron con una menor prevalencia de anemia y deficiencia de vitamina A. Las políticas públicas enfocadas en los grupos geográfica y socialmente vulnerables son necesarias para promover la equidad.

## Introduction

Anemia and vitamin A deficiency in children are priority nutrition problems on the public nutrition policy agenda due to their historically high prevalence and severe health consequences. These consequences include growth deficit, poor cognitive performance, weakened immune system, and increased morbidity and mortality from infectious diseases ^1^ ^,^ ^2^ ^,^ ^3^.

Local studies conducted from 2003 to 2015 showed that several demographic and socioeconomic factors are associated with anemia and vitamin A deficiency prevalence in Brazilian children. These factors include poor socioeconomic conditions of the mother or caregiver (enrolled in a social benefit program, low education, younger age, and black or mixed-race), unfavorable household characteristics (such as the presence of more than one resident < 5 years old and poor sanitation), and household food insecurity ^4^ ^,^ ^5^ ^,^ ^6^ ^,^ ^7^ ^,^ ^8^ ^,^ ^9^ ^,^ ^10^. Furthermore, prolonged or exclusive breastfeeding for at least six months, higher consumption of non-dairy protein foods (meat, eggs, or both), foods that are sources of vitamin A, and provitamin carotenoids, and micronutrient supplementation have been identified as factors that reduce the occurrence of anemia and vitamin A deficiency ^11^ ^,^ ^12^.

Child’s age is another important factor associated, i.e., children < 24 months are at increased risk for anemia, due to the remarkable physical growth in this age group, which increases the need for iron and other micronutrients. Low gastric capacity, which limits the intake of iron-rich foods per meal, and greater susceptibility to infections that can affect a child’s nutritional status may also explain this greater vulnerability ^11^.

A comparison of results from the latest national household surveys of children < 5 years old shows a 50% decrease in the prevalence of anemia and a 65% decrease in vitamin A deficiency from 2006 to 2019 ^13^. Therefore, this study aimed to analyze the association of socioeconomic, demographic, and dietary factors, and micronutrient supplementation with anemia and vitamin A deficiency in Brazilian children < 5 years old by age group in 2019.

## Methods

**Figure 1:**
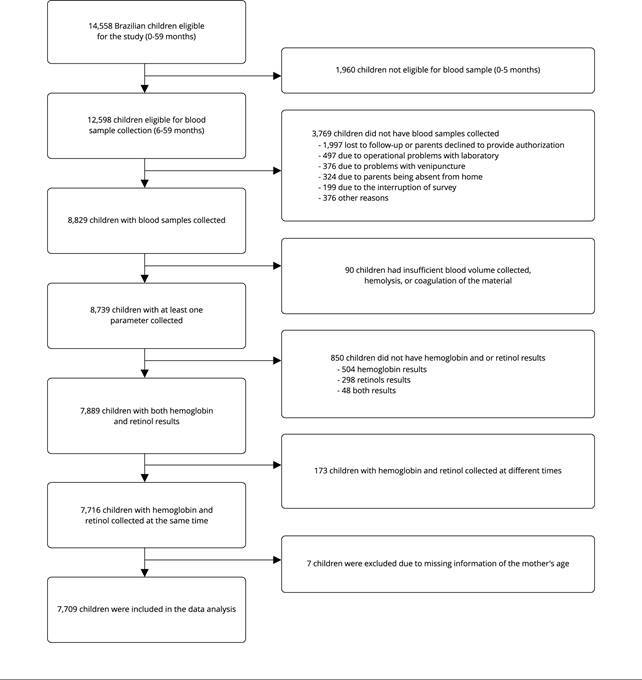
Data collection flowchart. *Brazilian National Survey on Child Nutrition* (ENANI-2019).

This study used data from ENANI-2019, a national household survey with a complex probabilistic sample ^14^ that examined 14,558 Brazilian children < 5 years old ^15^. In total, 12,598 children from 6-59 months were eligible for blood collection, of whom 8,829 had the biological material collected. Infants < 6 months of age were not included due to the greater difficulty with venipuncture and increased risk of post-blood draw complications (e.g., bruises), and the lack of reference values for diagnosing micronutrient deficiencies. The study included 7,716 children with data available for both hemoglobin (a biomarker of anemia status) and serum retinol (a biomarker of vitamin A status) ^14^. Seven children were excluded from the present analysis because no information on maternal age was available. The final sample comprised 7,709 children: 2,434 with 6-23 months of age and 5,275 with 24-59 months of age (Figure 1).

### Laboratory analyses and indicators of anemia and vitamin A deficiency

Blood collection and laboratory analysis were detailed in Castro et al. ^16^. Briefly, 8mL of blood was collected by venipuncture and divided between an EDTA tube (2mL) and a trace tube (6mL) wrapped in aluminum foil and stored in a cooler with a controlled temperature from 2ºC to 8ºC for transportation. After centrifugation of the trace tube, the serum was transferred to a second trace tube wrapped in aluminum foil to ensure sample stability for vitamin A analysis and stored at freezing temperature until the analyses. Firstly, vitamin A analysis was performed on the serum sample, due to its greater instability. Blood counts were performed on whole blood using a hematology analyzer with cell analysis by flow cytometry (UniCell DxH; https://www.beckmancoulter.com/). Serum vitamin A concentrations were determined by high-performance liquid chromatography (HPLC) with ultraviolet detection (HPLC Chromsystems; https://chromsystems.com/). These two methods are considered the gold standard for anemia and vitamin A deficiency diagnosis, respectively ^17^ ^,^ ^18^.

### Variables evaluated in the study

Anemia was classified as hemoglobin concentration < 11g/dL ^19^ and vitamin A deficiency was classified as serum retinol < 0.7μmol/L ^12^. 

**Figure 2:**
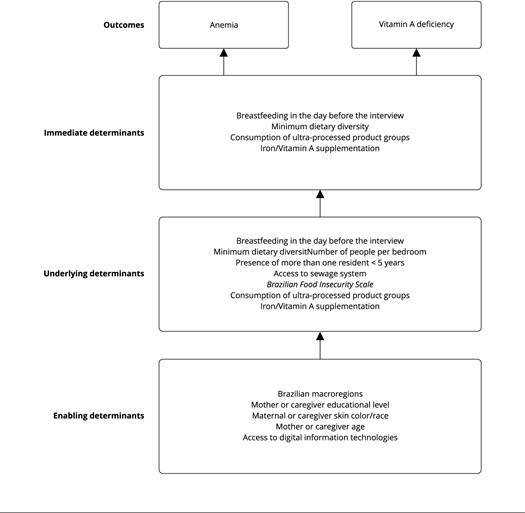
Conceptual framework on the determinants of maternal and child nutrition used in this analysis.

Note: the conceptual framework was based on the United Nations Children’s Fund (UNICEF) ^20^.

The independent variables used in the association analysis for both outcomes were selected based on the variables available in the database and according to a theoretical model on the determinants of all forms of malnutrition proposed by the United Nations Children’s Fund (UNICEF) ^20^ and the literature (Figure 2). The UNICEF model divides the determinants of malnutrition into three levels: (1) enabling determinants, which include political, financial, social, cultural, and environmental conditions; (2) underlying determinants, which include foods, services, and practices available to children and their families; and (3) immediate determinants, which are at the most proximal level and include diet and care that, when simultaneously available, provide children with adequate nutritional status ^20^.

In this study, the following variables were considered enabling determinants: Brazilian macroregions (North, Northeast, Southeast, South, and Central-West); maternal or caregiver years of schooling (0-7, 8-10, 11, ≥ 12 years); maternal or caregiver race/skin color (white, mixed-race, black); age of the mother or caregiver (< 20, 20-34, ≥ 35 years old); and access to digital information technologies: cell phone with access to internet, access to internet at home, and cable television (0, 1, 2-3 items). The options “yellow” (Asian descendants) and “indigenous people” were omitted from the child’s race/skin color analysis due to the low precision of estimates.

The underlying determinants evaluated included the number of people per bedroom (1, 2, 3, ≥ 4), the presence of more than one resident < 5 years of age in the household (yes, no), the availability of a sewage system (general or rainwater system, no), and the food insecurity situation of the household, assessed using the *Brazilian Food Insecurity Scale* [the Portuguese acronym EBIA - *Escala Brasileira de Insegurança Alimentar*] (security, mild insecurity, moderate insecurity, or severe insecurity) ^21^ ^,^ ^22^.

The immediate determinants evaluated included information on breastfeeding the day before the interview (yes; no - included only in the analyses of children < 2 years old), achievement of minimum dietary diversity (MDD) the day before the interview (consumption of foods from at least five of the following groups: breast milk; grains, roots, tubers, and plantains; pulses, nuts, and seeds; dairy products; flesh foods; eggs; vitamin A rich fruits and vegetables; other fruits and vegetables) ^23^, and consumption of ultra-processed food groups the day before (0, 1-2, ≥ 3), namely: soft drinks, other sugary beverages (industrially-produced juice, packaged juice, packaged coconut water, natural guarana or guarana syrup, redcurrant syrup, powdered juice, or natural fruit juice with added sugar); packaged snacks (including chips); sweet or savory biscuits/cookies; treats (candy, lollipop, other); yogurts; industrially produced bread (e.g., bread, loaf bread, hamburger bun); instant rice, corn, wheat, or oat flours; processed meats (hamburger, ham, mortadella, salami, nugget, sausage); and instant noodles. Moreover, micronutrient supplementation during the study or in the six months prior was evaluated: iron supplementation (yes or no, only in analyses with anemia as an outcome) and vitamin A supplementation (yes or no, only in analyses with vitamin A deficiency as an outcome).

### Data analysis

For data analysis, basic sample weights of children in the studied subset (n = 7,709) were adjusted considering predictor variables for non-response (absence of laboratory results for biomarkers) ^24^. 

Relative frequencies and the respective 95% confidence intervals (95%CI) of independent variables and outcomes were described. The prevalence of anemia and vitamin A deficiency was estimated according to independent variables. Coefficients of variation (CV), which is a relative measure of precision obtained by the ratio between the standard error and the estimated value of the indicator, were estimated to assess the precision of the estimates. For ENANI-2019, the CVs refer to prevalence. Estimates with a high CV may indicate that the sample is not large enough to make a population-level estimate with an acceptable precision. A CV under 30% was considered to have an appropriate precision for estimates produced in ENANI-2019 ^25^.

A hierarchical approach was adopted using UNICEF three levels model to assess factors associated with anemia and vitamin A deficiency. Quasi-Poisson models were constructed with binary, unadjusted (Model 1), and adjusted (Model 2) outcomes by age group (6-23, 24-59 months). Analyses were stratified by age group since younger children are more susceptible to micronutrient deficiencies ^11^ ^,^ ^24^ and due to differences between groups in dietary intake and micronutrient supplementation ^26^ ^,^ ^27^.

The magnitude of the association was estimated using the prevalence ratio (PR) with the respective 95%CI. The final fitted models were constructed so that the block of underlying determinants variables was adjusted by the variables of the most distal level (enabling determinants). The block of variables of the immediate determinants was adjusted by the variables of the preceding levels (underlying determinants and enabling determinants). Models 1 and 2, in which the outcome was anemia, were additionally adjusted for the variable child’s age expressed in a continuous format. 

All variables from each level were used to adjust the next level. Furthermore, models were tested using only variables with statistically significant associations for adjustment, and the results were similar to the selected approach.

All hypothesis tests were evaluated at a 5% significance level. Analyses were performed incorporating the survey sample design using the *survey* and *svyglm* packages of the R (http://www.r-project.org) programming language.

### Ethical considerations

The ENANI-2019 was approved by the Research Ethics Committee of the Clementino Fraga Filho University Hospital of the Federal University of Rio de Janeiro (UFRJ; CAAE n. 89798718.7.0000.5257). Data were collected after a parent or caregiver of the child authorized participation in the study through informed consent form.

## Results

Table 1 shows the distribution of socioeconomic, demographic and dietary factors, and micronutrient supplementation for the total number of children by age group.

**Table 1:** Distribution of socioeconomic, demographic and dietary factors, and supplementation by age group. *Brazilian National Survey on Child Nutrition* (ENANI-2019).

Variables	6-59 months (n = 7,709)		6-23 months (n = 2,434)		24-59 months (n = 5,275)	
	%	95%CI	%	95%CI	%	95%CI
Enabling determinants						
Brazilian macroregions						
Southeast	39.3	39.3; 39.3	39.1	39.1; 39.2	39.3	39.3; 39.4
North	10.9	10.9; 10.9	11.0	11.0; 11.0	10.9	10.9; 10.9
Northeast	28.1	28.1; 28.1	28.2	28.2; 28.2	28.1	28.0; 28.1
South	13.4	13.4; 13.5	13.4	13.4; 13.4	13.4	13.4; 13.5
Central-West	8.3	8.3; 8.3	8.3	8.3; 8.3	8.3	8.3; 8.3
Mother or caregiver educational level (completed years)						
0-7	23.5	21.3; 25.8	22.2	18.8; 25.6	24.2	21.7; 26.7
8-10	21.8	19.5; 24.0	23.8	20.3; 27.4	20.7	18.3; 23.1
11	37.5	35.0; 39.9	37.1	33.2; 40.9	37.7	34.7; 40.6
≥ 12	17.2	14.9; 19.6	16.9	13.2; 20.7	17.4	14.9; 19.9
Maternal or caregiver race/skin color						
White	30.9	28.0; 33.7	31.0	26.7; 35.3	30.8	27.8; 33.8
Mixed-race	55.8	52.1; 59.5	55.0	50.9; 59.2	56.2	51.9; 60.4
Black	12.3	10.3; 14.2	12.8	9.8; 15.7	12.0	9.7; 14.4
Yellow/Indigenous	1.1 *	0.4; 1.7	1.2 *	0.3; 2.0	1.0 *	0.3; 1.8
Access to digital information technologies (number of items) **						
0	7.1	5.8; 8.4	6.6	4.7; 8.5	7.3	5.9; 8.7
1	31.0	27.6; 34.5	30.8	26.3; 35.3	31.1	27.1; 35.1
2-3	61.9	58.2; 65.6	62.6	57.8; 67.3	61.6	57.4; 65.7
Mother or caregiver age (years)						
< 20	6.3	5.2; 7.5	10.8	8.7; 13.0	4.1	2.9; 5.2
20-34	66.8	64.2; 69.3	69.0	64.9; 73.2	65.7	63.1; 68.2
≥ 35	26.9	24.5; 29.3	20.1	16.8; 23.5	30.3	27.6; 32.9
Underlying determinants						
Number of people per bedroom						
1	4.9	3.9; 5.9	4.4	2.9; 5.9	5.2	4.0; 6.3
2	50.8	47.3; 54.3	47.3	43.8; 50.8	52.6	48.6; 56.5
3	28.2	25.7; 30.6	31.9	28.6; 35.3	26.3	23.6; 29.0
≥ 4	16.1	13.6; 18.6	16.4	13.8; 18.9	16.0	13.2; 18.7
Presence of more than one resident < 5 years of age						
Yes	29.1	25.8; 32.3	30.2	26.2; 34.1	28.5	25.0; 32.0
Sewage system						
Yes	72.9	69.4; 76.3	70.9	66.5; 75.2	73.9	70.4; 77.4
*Brazilian Food Insecurity Scale*						
Security	51.6	45.4; 57.8	53.2	46.1; 60.2	50.8	44.7; 56.9
Mild insecurity	37.8	32.3; 43.4	36.6	30.3; 42.8	38.5	32.8; 44.2
Moderate insecurity/Severe insecurity	10.6	8.8; 12.4	10.2	8.4; 12.1	10.7	8.7; 12.8
Immediate determinants						
Breastfeeding in the previous day ***						
Yes	24.4	22.8; 26.1	52.2	48.2; 56.3	-	-
Minimal dietary diversity						
Yes	61.3	58.2; 64.4	64.1	60.6; 67.5	60.0	56.2; 63.8
Vitamin A supplement use ^#^						
Yes	36.1	31.1; 41.2	45.9	39.5; 52.3	31.2	26.1; 36.4
Iron supplement use ^#^						
Yes	20.7	18.3; 23.1	33.7	30.4; 37.0	14.2	11.1; 17.3
Consumption of ultra-processed foods (groups)						
0	10.3	8.8; 11.7	18.6	15.7; 21.5	6.1	4.8; 7.4
1-2	46.4	42.9; 49.9	47.6	43.6; 51.6	45.7	41.7; 49.8
≥ 3	43.4	39.2; 47.5	33.8	28.9; 38.8	48.2	43.6; 52.7

95%CI: 95% confidence interval.

* Coefficients of variation > 30%, a measure of dispersion indicating the heterogeneity of the data;

** Access to technology items: smartphone with access to the internet; access to the internet at home; cable TV;

*** Consumption of breast milk on the day before the interview;

^#^ Supplementation during the study period or in the 6 months preceding it.

In 2019, the prevalence of anemia in children 6-59 months in Brazil was 10.3% (95%CI: 8.1; 12.5), 19.9% (95%CI: 16.4; 23.3) in children 6-23 months, and 5.6% in children 24-59 months (95%CI: 3.5; 7.6). The prevalence of vitamin A deficiency in children 6-59 months in Brazil was 6.4% (95%CI: 5.5; 7.4), with no statistical differences between prevalence by age group (7%; 95%CI: 5.5; 8.5) vs. 6.1% (95%CI: 5.1; 7.2).

### Factors associated with anemia in children 6-23 months

We observed a higher prevalence of anemia in children 6-23 months in the North Region (29.9%), in children whose mothers had up to seven years of schooling (28%), were mixed-race (24.2%), or were < 20 years old (34.2%); in those without access to digital information technologies in their households (27.5%), whose household had ≥ 4 people per bedroom (26.4%), more than one resident < 5 years old (29%), did not have a sewage system (23.8%), had moderate or severe food insecurity (27.3%); in those who had not received micronutrient supplementation (21.1%); and in those who had breastfed on the day before the interview (24.1%), had not reached minimum dietary diversity (24.9%), or had not consumed ultra-processed foods (22%) (Table 2).

**Table 2:** Prevalence (%), prevalence ratio (PR), and 95% confidence interval (95%CI) of anemia regarding factors assessed by age group in Brazilian children 6-59 months. *Brazilian National Survey on Child Nutrition* (ENANI-2019).

Variables	6-23 months					24-59 months				
	%	Model 1 *		Model 2 **		%	Model 1 *		Model 2 **	
		PR	95%CI	PR	95%CI		PR	95%CI	PR	95%CI
Enabling determinants										
Brazilian macroregion										
Southeast	18.2	Reference		Reference		3.1 ***	Reference		Reference	
North	29.9	1.64	1.13; 2.37	1.40	0.99; 1.99	10.6	3.42	1.58; 7.39	3.11	1.58; 6.13
Northeast	21.3	1.18	0.70; 1.98	1.13	0.67; 1.91	8.3 ***	2.71	0.99; 7.44	2.44	0.87; 6.83
South	14.0	0.77	0.50; 1.20	0.92	0.57; 1.47	3.5	1.15	0.51; 2.61	1.11	0.46; 2.69
Central-West	18.7	1.04	0.70; 1.55	0.99	0.67; 1.48	4.7	1.51	0.72; 3.14	1.43	0.71; 2.87
Educational level of mother or caregiver (completed years)										
0-7	28.0	2.26	1.41; 3.62	1.92	1.10; 3.34	6.7	2.51	1.18; 5.36	1.59	0.67; 3.79
8-10	22.1	1.80	1.21; 2.68	1.56	0.98; 2.47	7.1	2.46	1.03; 5.89	1.79	0.65; 4.94
11	17.1	1.41	0.88; 2.27	1.25	0.72; 2.17	5.2	1.87	0.76; 4.59	1.51	0.58; 3.94
≥ 12	12.1	Reference		Reference		2.8 ***	Reference		Reference	
Maternal or caregiver race/skin color						
White	13.1	Reference		Reference		4.6	Reference		Reference	
Mixed-race	24.2	1.83	1.30; 2.58	1.57	1.06; 2.33	5.6	1.21	0.75;1.95	0.81	0.51; 1.29
Black	18.0	1.40	0.84; 2.32	1.17	0.68; 2.02	8.0	1.74	0.95; 3.19	1.42	0.84; 2.40
Access to digital information technologies (number of items) ^#^										
0	27.5	1.62	1.02; 2.58	1.26	0.76; 2.08	9.1	2.23	1.26; 3.93	1.67	0.88; 3.18
1	23.4	1.36	1.03; 1.80	1.06	0.78; 1.43	7.8	1.93	1.41; 2.64	1.48	1.11; 1.97
2-3	17.3	Reference		Reference		4.0	Reference		Reference	
Age of mother or caregiver (years)										
< 20	34.2	2.88	1.56; 5.32	2.47	1.34; 4.56	13.7	2.33	1.04; 5.25	1.84	0.86; 3.95
20-34	20.4	1.80	1.04; 3.11	1.95	1.11; 3.44	5.3	1.02	0.62; 1.66	0.95	0.55; 1.62
≥ 35	10.4	Reference		Reference		5.0 ***	Reference		Reference	
Underlying determinants						
Number of people per bedroom						
1	14.0 ***	Reference		Reference		5.2 ***	Reference		Reference	
2	16.3	0.95	0.51; 1.78	0.83	0.45; 1.54	3.5	0.66	0.24; 1.82	0.56	0.21; 1.49
3	22.6	1.35	0.72; 2.53	0.95	0.50; 1.82	7.7	1.44	0.52; 3.97	1.02	0.36; 2.87
≥ 4	26.4	1.57	0.81; 3.04	0.94	0.43; 2.04	8.8	1.64	0.63; 4.28	1.00	0.37; 2.71
Presence of more than one resident aged under < 5 years						
No	15.9	Reference		Reference		4.4	Reference		Reference	
Yes	29.0	1.78	1.33; 2.39	1.61	1.15; 2.25	8.5	2.02	1.30; 3.16	1.61	1.00; 2.60
Sewage system										
Yes	18.3	Reference		Reference		4.6	Reference	Reference	
No	23.8	1.26	0.93; 1.72	1.05	0.77; 1.43	8.3	1.84	1.30; 2.62	1.16	0.88; 1.52
*Brazilian Food Insecurity Scale*										
Security	18.4	Reference		Reference		3.9	Reference		Reference	
Mild insecurity	19.9	1.09	0.80; 1.49	0.96	0.72; 1.29	5.9	1.51	0.99; 2.30	1.22	0.80; 1.84
Moderate insecurity/Severe insecurity	27.3	1.51	0.93; 2.45	1.03	0.60; 1.75	11.8	2.94	1.93; 4.49	1.50	0.92; 2.42
Immediate determinants										
Breastfeeding in the previous day ^##^										
Yes	24.1	Reference		Reference		-	Reference		Reference	
No	15.2	0.73	0.49; 1.09	0.71	0.49; 1.02	-	-	-	-	-
Minimal dietary diversity										
Yes	17.1	Reference		Reference		4.8	Reference		Reference	
No	24.9	1.29	0.91; 1.82	1.20	0.86; 1.68	6.7	1.47	1.00; 2.18	1.16	0.78; 1.73
Consumption of ultra-processed foods (groups)										
0	22.0	Reference		Reference		10.3	Reference		Reference	
1-2	20.1	1.14	0.73; 1.78	1.20	0.81; 1.78	3.5	0.33	0.17; 0.65	0.44	0.25; 0.81
≥ 3	18.3	1.21	0.71; 2.05	1.20	0.70; 2.07	6.9	0.68	0.35; 1.32	0.81	0.49; 1.35
Iron supplement use ^###^										
Yes	17.5	Reference		Reference		4.7	Reference		Reference	
No	21.1	1.19	0.86; 1.65	1.04	0.75; 1.45	5.7	1.28	0.68; 2.41	1.23	0.68; 2.22

Notes: bold values indicate statistically significant associations. Prevalence and PR estimates were considered statistically significant when no overlapping of 95%CIs was observed. Breastfeeding was not included in the model for children aged ≥ 24 months. The estimates for yellow/indigenous race/skin color were omitted from the table due to the low precision of these results (coefficientes of variation - CV > 30%).

* Model 1: adjusted by child age in months (continuous);

** Model 2: the adjustment was performed within each level by variables of the previous levels + child age in months (continuous);

*** CV > 30%;

^#^ Access to technology items: smartphone with access to the internet; access to the internet at home; cable TV;

^##^ Consumption of breast milk on the day before the interview;

^###^ Supplementation during the study period or in the six months preceding it.

In the adjusted model, educational level, maternal or caregiver race/skin color, and maternal or caregiver age were associated with anemia at the level of enabling determinants. The prevalence of anemia was 92% higher in children whose mothers had up to seven years of schooling (PR = 1.92; 95%CI: 1.10; 3.34) compared with those whose mothers had 12 years of schooling; 57% higher among those whose mothers were mixed-race (PR = 1.57; 95%CI: 1.06; 2.23) compared with those whose mothers were white; higher among those whose mothers < 20 years (PR = 2.47; 95%CI: 1.34; 4.56) and from 20-34 years old (PR = 1.95; 95%CI: 1.11; 3.44), compared with children whose mothers were ≥ 35 years (Table 2).

At the level of underlying determinants, the prevalence of anemia was 61% higher in children living with another child < 5 years old (PR = 1.61; 95%CI: 1.15; 2.25), than in children who did not live with another child of this age range. Variables at the immediate determinants level were not statistically associated with anemia in children under two years old (Table 2).

### Factors associated with anemia in children 24-59 months

We observed a higher prevalence of anemia in children 24-59 months in the North Region (10.6%), those whose mothers had from 8-10 years of schooling (7.1%), were black (8%) or were < 20 years old (13.7%); those without access to digital information technologies in their households (9.1%), had ≥ 4 people per bedroom (8.8%), had more than one resident < 5 years old (8.5%), did not have a sewage system available (8.3%), who had moderate or severe food insecurity (11.8%); who had not received iron micronutrient supplementation (5.7%); and who, on the day before the interview, had not reached MMD (6.7%) or had not consumed ultra-processed foods (10.3%).

In the adjusted model, Brazilian macroregions and access to digital information technologies were associated with anemia at the level of enabling determinants. Anemia was more prevalent in children 24-59 months living in the North Region than among those living in the Southeast (PR = 3.11; 95%CI: 1.58; 6.13). Moreover, anemia was 48% more prevalent among those who had access to only one of the technology items analyzed in their households (PR = 1.48; 95%CI: 1.11; 1.97) than among those who had access 2-3 items in their households (Table 2). 

At the level of underlying determinants, no variables were associated with anemia in children 24-59 months. At the level of immediate determinants, anemia was 56% lower in children who consumed 1-2 ultra-processed foods than in children who had not consumed ultra-processed foods the day before the interview (PR = 0.44; 95%CI: 0.25; 0.81) (Table 2).

### Factors associated with vitamin A deficiency in children 6-23 months

We observed a higher prevalence of vitamin A deficiency in children 6-23 months in the Central-West Region (12.2%), those whose mothers had up to seven years of schooling (9.3%), were mixed-race (7.6%), or were ≥ 35 years old (7.5%); whose households did not have access to digital information technologies (9.9%), had ≥ 4 people per bedroom (8.6%), had more than one resident < 5 years (10.5%), had no sewage system (8.3%), had moderate or severe food insecurity (12.6%); those who had received vitamin A supplementation (7.1%); and those who had been breastfed in the previous day (8.4%), had not reached the MDD (9.1%), or had consumed 1-2 ultra-processed foods (7.2%).

In the adjusted model, the factors associated with vitamin A deficiency at the level of enabling determinants were Brazilian macroregions and access to digital information technologies. The prevalence of vitamin A deficiency was higher among children living in the Central-West (PR = 2.32; 95%CI: 1.33; 4.05) than among children living in the Southeast; and 54% lower among children that had access to one technology item analyzed in their households (PR = 0.54; 95%CI: 0.33; 0.87) than among children who had access to 2-3 technology items analyzed in their households (Table 3).

**Table 3:** Prevalence (%), prevalence ratio (PR), and 95% confidence interval (95%CI) of vitamin A deficiency regarding factors assessed by age group in Brazilian children 6-59 months. *Brazilian National Survey on Child Nutrition* (ENANI-2019).

Variables	6-23 months					24-59 months				
	%	Model 1 *		Model 2 **		%	Model 1 *		Model 2 **	
		PR	95%CI	PR	95%CI		PR	95%CI	PR	95%CI
Enabling determinants										
Brazilian macroregions										
Southeast	5.6	Reference		Reference		4.2	Reference		Reference	
North	7.6	1.36	0.81; 2.28	1.48	0.86; 2.56	8.8	2.10	1.24; 3.56	1.96	1.16; 3.30
Northeast	6.4	1.15	0.60; 2.19	1.08	0.57; 2.05	4.9	1.18	0.73; 1.91	1.15	0.70; 1.88
South	8.7 ***	1.54	0.73; 3.26	1.50	0.67; 3.35	11.1	2.67	1.60; 4.44	3.07	1.89; 5.01
Central-West	12.2	2.17	1.26; 3.74	2.32	1.33; 4.05	8.1	1.94	1.14; 3.33	1.91	1.12; 3.25
Mother or caregiver educational level (completed years)										
0-7	9.3	1.39	0.65; 2.95	1.73	0.69; 4.33	6.0	1.22	0.73; 2.03	1.16	0.73; 1.84
8-10	7.9	1.17	0.56; 2.46	1.41	0.63; 3.18	7.8	1.58	0.96; 2.58	1.44	0.91; 2.27
11	5.2	0.78	0.35; 1.71	0.86	0.37; 2.01	5.9	1.20	0.73; 1.98	1.16	0.72; 1.87
≥ 12	6.7 ***	Reference		Reference		4.9	Reference		Reference	
Maternal or caregiver race/skin color										
Whhite	6.9	Reference		Reference		5.4	Reference		Reference	
Brown	7.6	1.10	0.60; 2.01	1.18	0.61; 2.25	6.4	1.19	0.81; 1.75	1.42	0.99; 2.05
Black	5.3 ***	0.77	0.28; 2.10	0.81	0.28; 2.40	6.7	1.24	0.65; 2.35	1.50	0.80; 2.81
Access to digital information technologies (number of items) ^#^										
0	9,9 ***	1.29	0.60; 2.75	0.97	0.40; 2.36	5.8 ***	0.95	0.42; 2.12	0.95	0.43; 2.07
1	5.0	0.66	0.42; 1.04	0.54	0.33; 0.87	6.4	1.05	0.71; 1.53	0.91	0.60; 1.39
2-3	7.7	Reference		Reference		6.1	Reference		Reference	
Mother or caregiver age (years)										
< 20	7.0 ***	0.93	0.37; 2.37	0.87	0.33; 2.29	5.8 ***	1.42	0.64; 3.15	1.20	0.57; 2.56
20-34	6.9	0.92	0.52; 1.64	0.99	0.56; 1.75	7.1	1.73	1.18; 2.52	1.62	1.11; 2.35
≥ 35	7.5	Reference		Reference		4.1	Reference		Reference	
Underlying determinants										
Number of people per bedroom										
1	7.0 ***	Reference		Reference		3.7 ***	Reference		Reference	
2	6.8	0.97	0.45; 2.08	0.99	0.41; 2.43	5.6	1.51	0.75; 3.06	1.44	0.73; 2.85
3	6.5	0.92	0.39; 2.13	0.79	0.31; 2.03	7.4	2.01	0.87; 4.67	2.02	0.92; 4.43
≥ 4	8.6	1.22	0.50; 2.97	0.95	0.30; 2.97	6.6	1.79	0.89; 3.60	1.72	0.84; 3.49
Presence of more than one resident < 5 years of age										
No	5.5	Reference		Reference		5.7	Reference		Reference	
Yes	10.5	1.90	1.15; 3.13	1.82	1.09; 3.05	7.3	1.28	0.87; 1.87	1.14	0.80; 1.63
Sewage system										
Yes	6.5	Reference		Reference		6.3	Reference		Reference	
No	8.3	1.27	0.77; 2.11	1.14	0.69; 1.90	5.8	0.93	0.59; 1.45	0.66	0.43; 1.02
*Brazilian Food Insecurity Scale*										
Security	6.5	Reference		Reference		5.8	Reference		Reference	
Mild insecurity	6.2	0.94	0.55; 1.61	0.96	0.57; 1.64	6.5	1.11	0.75; 1.63	1.08	0.73; 1.59
Moderate insecurity/Severe insecurity	12.6	1.93	1.01; 3.68	1.84	0.89; 3.81	6.5	1.11	0.66; 1.86	0.99	0.53; 1.84
Immediate determinants										
Breastfeeding in the previous day ^##^										
Yes	8.4	Reference		Reference		-	Reference		Reference	
No	5.5	0.66	0.43; 1.02	0.56	0.34; 0.93	-	-	-	-	-
Minimal dietary diversity										
Yes	5.8	Reference		Reference		5.4	Reference		Reference	
No	9.1	1.57	0.93; 2.63	1.76	0.94; 3.31	7.3	1.36	0.98; 1.89	1.29	0.95; 1.75
Consumption of ultra-processed foods (groups)										
0	6.8	Reference		Reference		5.4	Reference		Reference	
1-2	7.2	1.05	0.57; 1.94	1.57	0.79; 3.10	6.0	1.11	0.65; 1.89	1.22	0.72; 2.08
≥ 3	6.9	1.01	0.55; 1.84	1.57	0.71; 3.46	6.4	1.19	0.64; 2.21	1.24	0.67; 2.30
Vitamin A supplement use ^###^										
Yes	7.1	Reference		Reference		6.1	Reference		Reference	
No	7.0	0.99	0.61; 1.59	0.91	0.56; 1.50	6.2	1.01	0.71; 1.44	0.97	0.67; 1.39

Notes: bold values indicate statistically significant associations. Prevalence and PR estimates were considered statistically significant when no overlapping of 95%CIs was observed. Breastfeeding was not included in the model for children aged ≥ 24 months. The estimates for yellow/indigenous race/skin color were omitted from the table due to the low precision of these results (coefficientes of variation - CV > 30%).

* Model 1: unadjusted;

** Model 2: each level was adjusted based on variables of the previous levels;

*** CV > 30%;

^#^ Access to technology items: smartphone with access to the internet; access to the internet at home; cable TV;

^##^ Consumption of breast milk on the day before the interview;

^###^ Supplementation during the study period or in the six months preceding it.

At the level of underlying determinants, the prevalence of vitamin A deficiency was 82% higher in children living with more than one resident < 5 years old (PR = 1.82; 95%CI: 1.09; 3.05) than in children who did not live with another child in this age group (Table 3). At the level of immediate determinants, non-breastfed children had a 44% lower prevalence of vitamin A deficiency (PR = 0.56; 95%CI: 0.34; 0.93) compared with breastfed children (Table 3).

### Factors associated with vitamin A deficiency among children 24-59 months

We found a higher prevalence of vitamin A deficiency in children 24-59 months in the Southern Region (11.1%), those whose mothers had from eight to 10 years of schooling (7.8%), were black (6.7%), or were 20-34 years (7.1%); those with access to only one digital information technology in their households (6.4%), a household with 3 people per bedroom (7.4%), a household with more than one resident < 5 years (7.3%), had access to sewage system (6.3%), had mild (6.5%) and moderate or severe (6.5%) food insecurity; those who had not received vitamin A supplementation (6.2%); and those who had not reached MDD the day before the interview (7.3%) or had consumed ≥ 3 ultra-processed foods (6.4%).

In the adjusted model, at the level of enabling determinants, children 24-59 months living in the North, South, and Central-West had a higher prevalence of vitamin A deficiency (PR = 1.96; 95%CI: 1.16; 3.30, PR = 3.07; 95%CI: 1.89; 5.01, and PR = 1.91; 95%CI: 1.12; 3.25, respectively) than children living in the Southeast. The prevalence of vitamin A deficiency was 62% higher among those whose mothers were 20-34 years (PR = 1.62; 95%CI: 1.11; 2.35) than among those whose mothers were ≥ 35 years old. All variables analyzed in the other levels were not statistically associated with vitamin A deficiency in children in this age group (Table 3).

## Discussion

Our findings show that social vulnerabilities are more frequently associated with anemia and vitamin A deficiency in children than dietary marker variables. Contextual factors (enabling determinants) were the main determinants of anemia. Regarding anemia, we found differences between maternal or caregiver educational level, age, and race/skin color in children 6-23 months. Furthermore, we found differences between Brazilian macroregions and levels of access to technology items in children 24-59 months. Regarding vitamin A deficiency, we found differences between macroregions for the two age groups, the levels of access to technology items only for the group of children 6-23 months, and the maternal or caregiver age groups for children 24-59 months.

Among the underlying determinants, the presence of more than one resident < 5 years old in the household was associated with a higher prevalence of anemia and vitamin A deficiency in children 6-23 months. Among the immediate determinants, only consumption of ultra-processed food groups and absence of breast milk on the day before the interview were inversely associated with anemia (children 24-59 months) and vitamin A deficiency (children 6-23 months). These results highlight the need for specific public policies for different regions of Brazil and focus on the most vulnerable social groups.

Among the enabling determinants of anemia in children 6-23 months, mixed-race maternal or caregiver was directly associated with anemia; however, maternal or caregiver age and maternal or caregiver educational level were inversely associated with this outcome (the younger the age and the lower the educational level, the greater this association). The inverse association between maternal educational level and anemia corroborates a previous study of children 6-60 months ^5^. However, the inverse association between maternal age and anemia in this study has not been previously found ^5^ ^,^ ^8^ ^,^ ^28^. Furthermore, different from our study, no direct association between maternal or caregiver race/skin color and child anemia has been previously observed ^8^. An inverse association was observed between maternal age and vitamin A deficiency in children 24-59 months, differing from the results found for the *Brazilian National Survey of Demograpy and Health* conducted in 2006 (PNDS 2006), which reported an association between maternal age of 36-50 years and the occurrence of vitamin A deficiency ^9^. Notably, the observed association between macroregions and anemia/vitamin A deficiency has not been previously explored, as well as the association between access to digital information technologies and anemia/vitamin A deficiency.

The presence of another child < 5 years old in the household was an essential factor associated with anemia (PR = 1.61) and vitamin A deficiency (PR = 1.82) in children < 24 months of age. Thus, greater need for care and nutrition due to the presence of more than one resident < 5 years old in the household makes families even more vulnerable regarding general living conditions. Silva et al. ^29^ observed a 67% higher prevalence of anemia in children 11-15 months living at home with another child < 5 years in four Brazilian cities (Rio Branco - Acre State, Olinda - Pernambuco State, Goiânia - Goiás State, and Porto Alegre - Rio Grande do Sul State) between 2012 and 2013. Castro et al. ^7^ observed a 27% higher prevalence of anemia among those who had siblings < 5 years, based on information from a cross-sectional population-based study in children 6-60 months in two cities in Acre in 2002.

Two proximal factors must be highlighted. First, consumption of 1-2 ultra-processed foods is associated with a lower prevalence of anemia in children ≥ 24 months of age compared with those who do not consume ultra-process products. It could be hypothesized that the consumption of ultra-processed foods consisting of iron-fortified wheat flour (since fortification is mandatory in the country) ^30^ and fortified with micronutrients could explain this result ^31^. However, ultra-processed foods have an imbalanced nutrient composition ^32^ ^,^ ^33^ and harm diet quality ^34^ ^,^ ^35^, thus they are contraindicated for children < 2 years old and should be avoided for this age group ^36^ ^,^ ^37^. A 2011-2012 study of 3-year-old children from low-income families in Southern Brazil (n = 432) found that those in the highest tertile of the distribution of ultra-processed foods consumption had a lower risk of anemia than those in the first tertile (odds ratio - OR = 0.56; 95%CI: 0.39; 0.82). On the other hand, higher levels of ultra-processed foods in the diet were associated with higher consumption of added sugars, total fats, and sodium and a lower intake of proteins, fiber, and calcium ^38^. Therefore, ensuring a diverse diet rich in micronutrient sources with good bioavailability is the best way to avoid anemia. Micronutrient supplementation must be offered only when necessary ^11^.

The second finding was that children 6-23 months who had not been breastfed the day before the interview had a lower prevalence of vitamin A deficiency than breastfed children, suggesting that breastfeeding is not a protective factor for vitamin A deficiency. However, we examined only continued breastfeeding in this study, which is more prevalent in groups with lower maternal education and lower socioeconomic status ^39^. This group with higher social vulnerability also has a lower prevalence of exclusive breastfeeding and lower MDD during complementary feeding ^26^ ^,^ ^39^ ^,^ ^40^. Furthermore, exclusively breastfed children until six months of age have higher serum concentrations of vitamin A ^41^ and a higher intake of this micronutrient during complementary feeding between 6-12 months of age ^29^. Therefore, we hypothesize that continued breastfeeding reflects differences in the pattern of exclusive breastfeeding and the introduction of complementary feeding. Future studies should examine this association, considering exclusive breastfeeding and indicators of diet quality as well as those discussed in this study as essential confounders. However, it is worth noting that breastfeeding is protective for several health outcomes and is recommended for at least 2 years and as exclusively feeding until 6 months of age ^37^.

Limitations of the study include the use of a qualitative assessment of dietary intake and breastfeeding on the day before the interview and supplementation, which precludes a quantitative evaluation of micronutrient supply, as well as lack of data on health status at birth (e.g., clamping [early or not] of the umbilical cord) and concurrent morbidities that might affect the nutritional status of iron and vitamin A.

Strengths of the study include the use of a theoretical model to guide data analysis; the wide range of variables available in the database, covering all levels of the model, and consequently the use of statistical modeling compatible with the theoretical model; the use of gold standard methods for the diagnosis of anemia and vitamin A deficiency ^16^ ^,^ ^42^, allowing greater precision in the estimation of these diseases; and large sample size, allowing for more precise estimates than those obtained in the PNDS 2006. This is the only Brazilian household survey prior to ENANI-2019, which collected blood data from children < 5 years of age and included 3,455 blood samples with a measure of hemoglobin and 3,499 of vitamin A, while this study included over 7,000 samples with both parameters. Furthermore, the PNDS 2006 used a different laboratory method, the dried blood spot method, which is not considered the gold standard for assessing anemia and vitamin A deficiency ^43^.

This study outcomes indicate the need for an approach to prevent and control anemia and vitamin A deficiency, considering the macroregional perspective and establishing mechanisms to prioritize the most vulnerable regions (North for anemia and vitamin A deficiency; Central-West for vitamin A deficiency). This approach, resulting from the ENANI-2019 findings, is already being implemented in the country by the Brazilian Ministry of Health and has led to the revision of prophylactic micronutrient supplementation programs aimed at controlling these two outcomes, such as the National Iron Supplementation Program and the National Vitamin A Supplementation Program ^44^. As a result, strategies have been developed to prioritize these macroregions in combination with other parameters related to the social vulnerability of families ^45^.

Furthermore, primary health care must pay special attention to children living with another child < 5 years old, those whose mothers had up to seven years of schooling, those whose mothers are adolescents, and those whose mothers or caregivers are mixed-race. Families in which these conditions overlap deserve more attention. This attention can be given, for example, by more regular monitoring that emphasizes dietary guidelines and micronutrient supplementation, and by the promotion of intersectoral actions of social support for these particularly vulnerable families.

This study showed that in an epidemiological scenario with a low prevalence of anemia and vitamin A deficiency in children < 5 years old in Brazil in 2019, sociodemographic and nutritional factors are associated with these diseases, indicating the need to prioritize public policies that promote health equity for this population group.
